# In Vitro and Ex Vivo Inhibition of Human Telomerase by Anti-HIV Nucleoside Reverse Transcriptase Inhibitors (NRTIs) but Not by Non-NRTIs

**DOI:** 10.1371/journal.pone.0047505

**Published:** 2012-11-15

**Authors:** Kyle R. Hukezalie, Naresh R. Thumati, Hélène C. F. Côté, Judy M. Y. Wong

**Affiliations:** 1 Genetics Graduate Program, The University of British Columbia, Vancouver, British Columbia, Canada; 2 Faculty of Pharmaceutical Sciences, The University of British Columbia, Vancouver, British Columbia, Canada; 3 Department of Pathology and Laboratory Medicine (HCFC), The University of British Columbia, Vancouver, British Columbia, Canada; University of Pittsburgh, United States of America

## Abstract

Telomerase is a specialized reverse transcriptase responsible for the de novo synthesis of telomeric DNA repeats. In addition to its established reverse transcriptase and terminal transferase activities, recent reports have revealed unexpected cellular activities of telomerase, including RNA-dependent RNA polymerization. This telomerase characteristic, distinct from other reverse transcriptases, indicates that clinically relevant reverse transcriptase inhibitors might have unexpected telomerase inhibition profiles. This is particularly important for the newer generation of RT inhibitors designed for anti-HIV therapy, which have reported higher safety margins than older agents. Using an in vitro primer extension assay, we tested the effects of clinically relevant HIV reverse transcriptase inhibitors on cellular telomerase activity. We observed that all commonly used nucleoside reverse transcriptase inhibitors (NRTIs), including zidovudine, stavudine, tenofovir, didanosine and abacavir, inhibit telomerase effectively in vitro. Truncated telomere synthesis was consistent with the expected mode of inhibition by all tested NRTIs. Through dose-response experiments, we established relative inhibitory potencies of NRTIs on in vitro telomerase activity as compared to the inhibitory potencies of the corresponding dideoxynucleotide triphosphates. In contrast to NRTIs, the non-nucleoside reverse transcriptase inhibitors (NNRTIs) nevirapine and efavirenz did not inhibit the primer extension activity of telomerase, even at millimolar concentrations. Long-term, continuous treatment of human HT29 cells with select NRTIs resulted in an accelerated loss of telomere repeats. All tested NRTIs exhibited the same rank order of inhibitory potencies on telomerase and HIV RT, which, according to published data, were orders-of-magnitude more sensitive than other DNA polymerases, including the susceptible mitochondria-specific DNA polymerase gamma. We concluded that telomerase activity could be inhibited by common NRTIs, including currently recommended RTI agents tenofovir and abacavir, which warrants large-scale clinical and epidemiological investigation of the off-target effects of long-term highly active antiretroviral therapy (HAART) with these agents.

## Introduction

Linear chromosomes are capped by telomeres, nucleoprotein structures that protect chromosome ends from nuclease digestion. Telomeres are comprised of simple DNA repeats and are packaged in a sequence-specific manner with the six-member protein complex known as shelterin [1]. Incomplete DNA replication at chromosome ends causes the loss of telomeric DNA with each cell division. Telomeric DNA loss is cumulative and is tolerated until telomeres reach a critically short length. When telomeres reach a critical length, cellular surveillance mechanisms are activated and cellular proliferation ceases, either by permanent cell-cycle arrest, known as senescence, or by apoptosis [2], [3].

Telomerase is a cellular reverse transcriptase responsible for the *de novo* synthesis of telomeric DNA repeats at the ends of linear chromosomes [4]. The catalytic core of the telomerase enzyme is a ribonucleoprotein composed of telomerase reverse transcriptase (TERT), the catalytic subunit [5], and telomerase RNA (TER) [6]. TERT uses a region of its integral RNA (TER) as a template for nucleotide addition. Based on the conservation of reverse transcriptase (RT) domain organization between HIV RT and TERT, several biochemical and cell biology studies were undertaken to characterize the inhibition profiles of HIV RT inhibitors. In immortalized human T- and B-lymphocyte cell lines, AZT, but not d4T, caused telomere shortening, and AZT-TP inhibited human telomerase *in vitro* [7]. The lack of observable d4T effects on telomere length might have been due to the inherently large variability in telomere length in the T- and B-lymphocyte cell lines. AZT has also been shown to inhibit telomerase and cause telomere shortening in human breast cancer cells [8], [9], colon cancer cells [10], and leukemia cells [11]. In addition, AZT-mediated telomerase inhibition was measured in a human hepatoma cell line [12], and telomere shortening was observed in cervical cancer cells [13]. The only other NRTI studied so far, Abacavir (ABC), inhibited telomerase in human meduloblastoma cells [14], [15]. The active form of ABC, CBV-TP, also inhibited human telomerase *in vitro*. Effects on telomere maintenance and telomerase activity of the adenosine analogs didanosine (ddI) and the newer NRTI, tenofovir (TFV) with a reported higher margin of safety, are not known. Likewise, there is currently no published data on the effects of NNRTIs nevirapine (NVP) and efavirenz (EFV) on human TERT catalysis.

**Figure 1:**
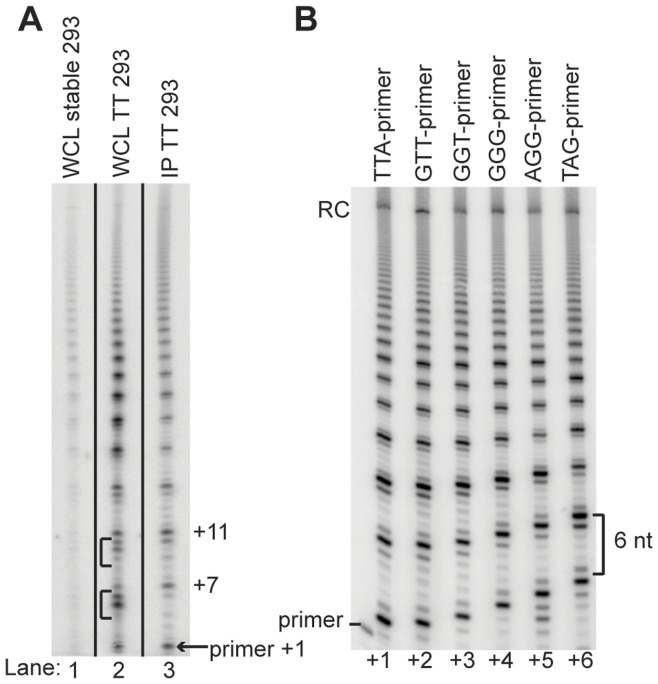
Optimization of the telomerase primer extension assay. **A.** Optimization of the primer extension assay for telomerase activity. Sources of telomerase activity: lane 1, whole cell lysate from 293HEK cells stably transfected with TERT and TER; lane 2, whole cell lysate from 293HEK cells transiently transfected with TERT and TER; lane 3, IP telomerase from 293HEK cells transiently transfected with 3×FLAG TERT and TER. Brackets in lane 2 denote DNA degraded by endogenous nucleases. **B.** IP telomerase activity in the presence of six different primers demonstrating that the primer extension assay is telomerase-specific. The DNA banding pattern with each primer can be predicted based on the template domain in TER. The number of nt added to each primer after first-repeat synthesis is shown below each lane.

**Table 1:** Summary of the relative potencies of selected NRTIs against telomerase catalysis *in vitro.*

Endogenous nucleotide	Inhibitor	Inhibitory Potency (IC_50_, µM)	Discrimination Factor*
**dTTP**	ddTTP	30.6	3.0
	d4T-TP	24.1	2.4
	AZT-TP	44.1	4.4
**dATP**	ddATP/ddI	12.6	0.63
	TFV-DP	109.7	5.5
**dGTP**	ddGTP	33.0	1.6
	CBV-TP	357	18

* Discrimination Factor (DF): fold increase in concentration of ddNTPs and NRTIs above respective endogenous competitor dNTP required for 50% telomerase inhibition *in vitro* (IC_50_).

[DF = (IC50_NRTI/ddNTP_)÷([dNTP_competitor_])].

**Figure 2:**
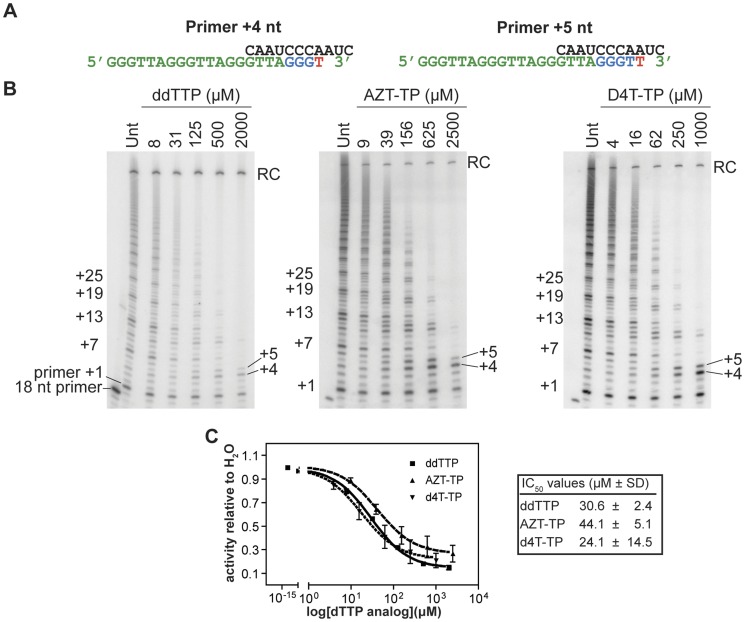
The thymidine analogs AZT and d4T inhibit telomerase *in vitro*. **A.** In the presence of increasing concentrations of ddTTP, d4T-TP, or AZT-TP (shown as T*), telomerase cannot synthesize past the primer +4 or primer +5 positions, and cannot translocate in order to synthesize further repeats. **B.** Representative gel images of IP telomerase activity in the presence of ddTTP, AZT-TP, or d4T-TP. Telomerase-specific DNA products are labeled on he left and right of each gel. Free, 18-nt end-labeled primer is shown for reference. **C.** Dose-response curves demonstrating telomerase inhibition by thymidine analogs. Solid line, ddTTP; long dashed line, AZT-TP; short dashed line, d4T-TP. Data shown were obtained from a minimum of three independent experiments. Error bars are mean ± SD. RC denotes recovery control.

**Figure 3:**
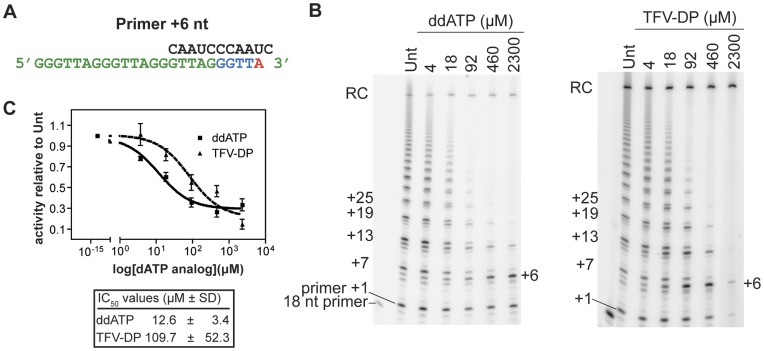
The adenosine analog TFV-DP inhibits telomerase *in vitro*. **A.** In the presence of increasing concentrations of ddATP or TFV-DP (shown as A*), telomerase cannot synthesize past the primer +6 position, and cannot translocate in order to synthesize further repeats. **B.** Representative gel images of IP telomerase activity in the presence of ddATP or TVF-DP. Telomerase-specific DNA products are labeled on the left and right of each gel. Free, 18-nt end-labeled primer is shown for reference. **C.** Dose-response curves demonstrating telomerase inhibition by adenosine analogs. Solid line, ddATP; dashed line, TFV-DP. Data were obtained from a minimum of three independent experiments per adenosine analog. Error bars are mean ± SD. RC denotes recovery control.

Telomerase performs essential cellular functions in human cells, and is the sole RT not associated with mobile genetic elements [16], [17]. Recent reports on the ability of TERT to support RNA-dependent RNA polymerization (RdRP) [18] and template-independent terminal transferase activity [19] set this enzyme apart from RTs commonly found in retroviruses and other mobile genetic elements. The reported RdRP activity suggests a role of TERT that is unrelated to telomere synthesis, perhaps important to its proposed extra-telomeric functions in carcinogenesis [18], [20]. The ability of TERT to add NTPs (RdRP), in addition to dNTPs (RT), to a 3′OH terminus implies a greater flexibility in the substrate binding pocket of this RT compared to other retrotransposon RTs in the human genome. The RdRP activity of TERT is associated with the enzyme’s intracellular trafficking to the mitochondria, perhaps as a response to oxidative stress [21], [22], [23]. While the exact mechanism is still elusive, TERT’s catalytic activity at the mitochondria may protect mtDNA against oxidative stress [23], [24], and prevent mitochondrial dysfunction [25] in a telomere-synthesis independent manner. To understand the substrate and catalysis properties of TERT, we undertook a biochemical study of the functional impact of an expanded spectrum of RT inhibitors on telomerase catalysis *in vitro* and in human cells.

## Materials and Methods

### Chemicals and Reagents

Aqueous solutions of D4T-triphosphate (TP) and AZT-TP were obtained from ChemCyte Inc. (San Diego, CA). Aqueous solutions of CBV-TP and TFV-DP were obtained from Moravek Biochemicals Inc. (Brea, CA, USA). Dideoxynucleotide triphosphates were obtained from MJS Biolynx (Brockville, ON). Nevirapine and efavirenz were obtained through the NIH AIDS reagent program. Nevirapine and efavirenz were dissolved in DMSO. Stock nucleotide analog concentrations were determined prior to every experiment through UV/visual spectrophotometry. All nucleotide analogs were added into the reaction as 20% of the final volume, while NVP and EFV were added as either 5% or 10% of the final volume.

All drugs for cell culture were obtained through The National Institutes of Health (NIH) AIDS Reagent Program (Germantown, MD).

**Figure 4:**
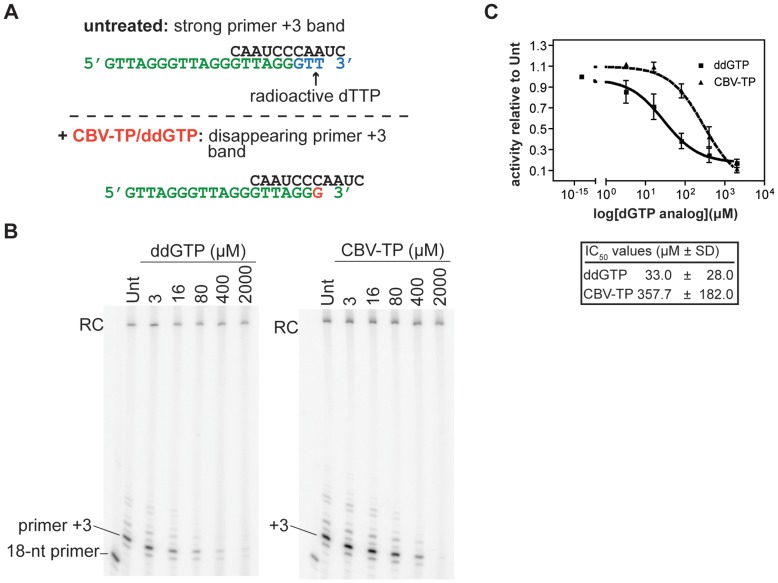
The guanosine analog CBV-TP inhibits telomerase *in vitro*. A. In the presence of increasing concentrations of ddGTP or CBV-TP (shown as G*), the radioactive signal at the primer +3 position diminishes. **B.** Representative gel images of IP telomerase activity in the presence of ddGTP or CBV-TP. Telomerase-specific DNA products are labeled on the left and right of each gel. Free, 18-nt end-labeled primer is shown for reference. **C.** Dose-response curves demonstrating telomerase inhibition by guanosine analogs. Solid line, ddGTP; dashed line, CBV-TP. Data were obtained from a minimum of three independent experiments per guanosine analog. Error bars are mean ± SD. RC denotes recovery control.

**Figure 5:**
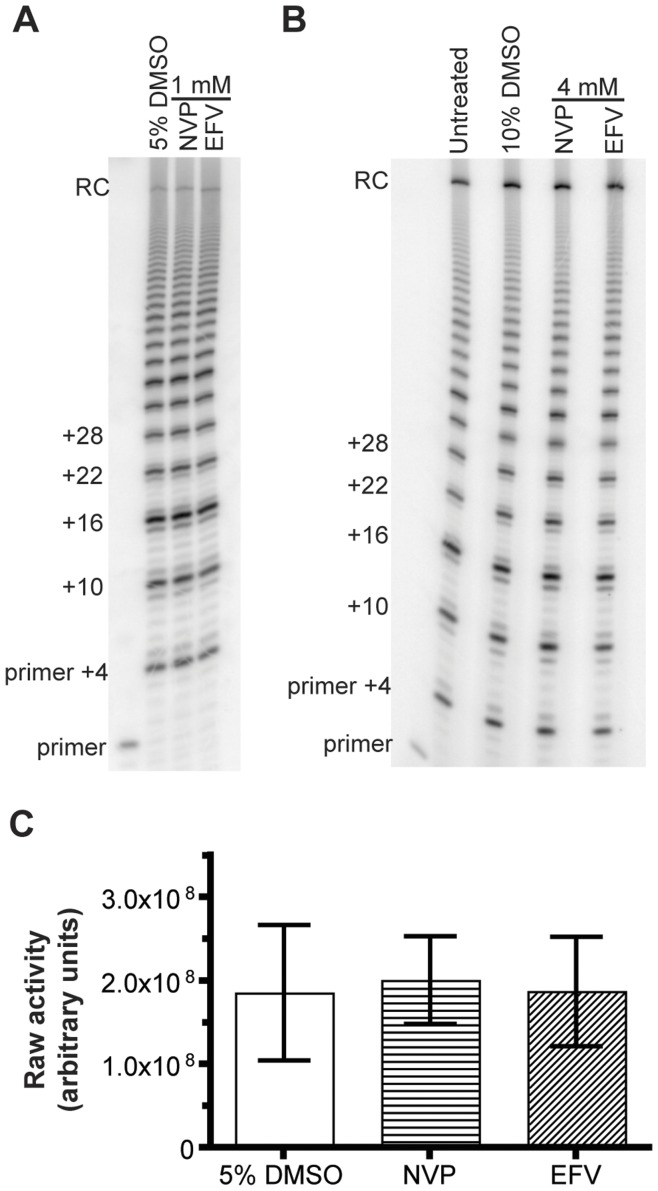
The NNRTIs NVP and EFV do not inhibit telomerase *in vitro*. Representative gel images showing telomerase activity in the presence of either 1 mM (**A**) or 4 mM (**B**) NVP or EFV. 5% DMSO was used as a vehicle control. **C.** Quantification of gel image shown in A. Data was obtained through the analysis of at least three independent experiments.

### Primer Extension Assay (Conventional Assay)

A typical reaction consisted of immunopurified telomerase (20 µL), dATP, dTTP, dGTP, either [α-^32^P]-labelled dGTP or dTTP, an 18 nt primer (2.5 µM), and assay buffer (50 mM Tris-acetate pH = 8.3, 1 mM MgCl_2_, 50 mM Potassium acetate, 1 mM spermidine, 5 mM β-mercaptoethanol). For thymidine analog experiments, [α-^32^P]dGTP (3000 Ci/mmol 10 mCi/mL, 3.3 µM, Perkin Elmer) was used, final nucleotide concentrations were 1 mM dATP, 10 µM dTTP, and 10 µM dGTP, and a primer with the permutation 5′-GGGTTAGGGTTAGGGTTA-3′ was used. The setup for adenosine analog experiments was identical except for the final dATP and dTTP concentrations, which were 20 µM and 1 mM, respectively. For guanosine analog experiments, [α-^32^P]dTTP (3000 Ci/mmol 10 mCi/mL, 3.3 µM, Perkin Elmer) was used. Final nucleotide concentrations were 1 mM dTTP, and 20 µM dGTP (dATP was withheld from the reaction), and a primer with the permutation 5′-GTTAGGGTTAGGGTTAGG-3′ was used. With NNRTIs, reactions were set up in the presence of [α-^32^P]dGTP, final nucleotide concentrations were 1 mM dATP, 1 mM dTTP, and 10 µM dGTP, and a primer with the permutation 5′-TTAGGGTTAGGGTTAGGG-3′ was tested. Reactions were incubated at 30°C for 2 h and stopped by 15 min incubation with Stop Solution A and B. After the addition of a 250-nt, ^32^P-end-labeled recovery control (RC), DNA products were PCI purified, precipitated and resolved on 17.5% (7 M urea) denaturing gels. Dried gels were exposed to a phosphor screen overnight. Images (100 µm resolution) were obtained using the Typhoon Trio Imager (GE Healthcare Life Sciences).

**Figure 6:**
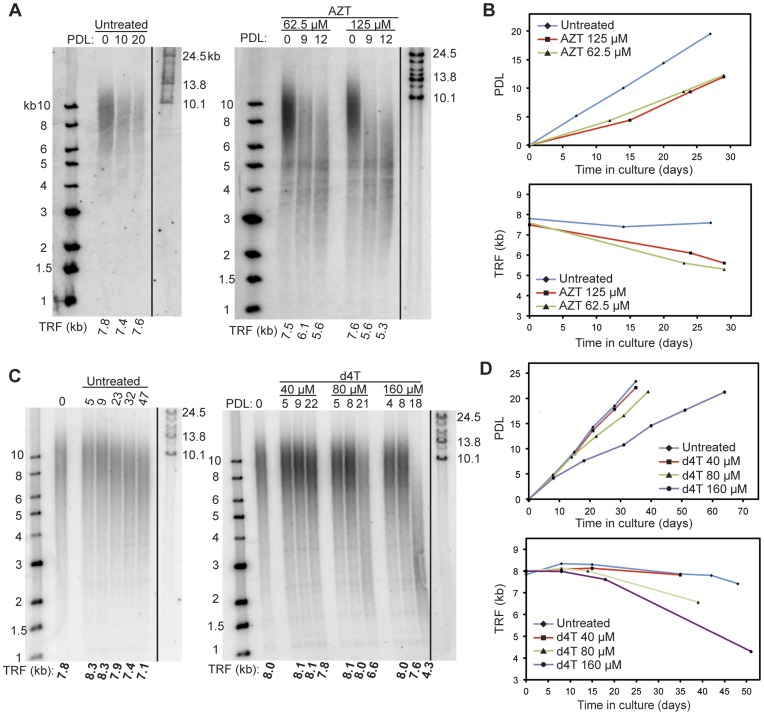
Continuous treatment of HT29 cells with the thymidine analog AZT and d4T causes substantial telomere shortening. A. TRF blots of untreated (left), AZT-treated (right) HT29 cells. PDL at which TRF was analyzed is shown above each lane. Molecular mass markers are shown at left and right of gel images. Each TRF smear was quantified as a weighted average and is shown below each lane. **B.** Growth curves and telomere maintenance dynamics of HT29 cells treated continuously with AZT. The growth curve and TRF dynamics of untreated HT29 cells (solid blue line) is plotted for comparison. Telomere length maintenance inhibition was observed in both the lower (62.5 µM) and higher (125 µM) AZT doses. **C.** TRF blots of untreated (left), d4T-treated (right) HT29 cells. **D.** Growth curves and telomere maintenance dynamics of HT29 cells treated continuously with d4T. The growth curve and TRF dynamics of untreated HT29 cells (solid blue line) is plotted for comparison. Loss of telomere length was observed with the middle (80 µM) and more significant at the higher (160 µM) d4T dose, but less evident in the lowest dose (40 µM) of d4T treatments, suggesting an apparent dose-response relationship.

**Table 2:** Summary of the impact of NRTIs/NNRTIs on telomere maintenance in HT29 colorectal adenocarcinoma cells.

	Drug	Plasma Cmax (µM)	Dose (µM)	TRF loss (kb)	TotalPDL
**Strong impact**					
	d4T	2–3	40	0.2	22
			80	1.4	21
			160	3.7	18
	AZT	5	62.5	1.9	12
			125	2.3	12
	ABC	10–15	12.5	0.9	19
			50	2.3	19
			100	0.7	2
**Moderate impact**					
	TDF	1	50	0.5	8
			100	0.6	3
	ddI	10	30	0.6	18
			60	0.8	18
			120	0.8	12
**No impact**					
	3TC	6–7	80	–	19
	NVP	20	100	–	19
			200	–	18
	EFV	12	6	–	20
			12	–	10

### Quantification of Telomerase Primer Extension Products

Quantification was performed using ImageQuant (v. 5.2, GE Healthcare Life Sciences) as follows. For experiments investigating thymidine analogs, guanosine analogs and ddATP, total enzyme activity for each reaction was determined by quantifying the signal of all bands between the primer +1 nt product and the RC. The total activity value was then normalized by dividing total activity by the primer +1 nt product for the respective lane. The activity-normalized values were then normalized to the untreated reaction (H_2_O) and plotted against the drug concentration to generate a dose-response curve for each experiment. For experiments testing TFV-DP and NNRTIs, quantification was essentially the same with the exception that total activity was not normalized to the primer +1 product.

**Figure 7:**
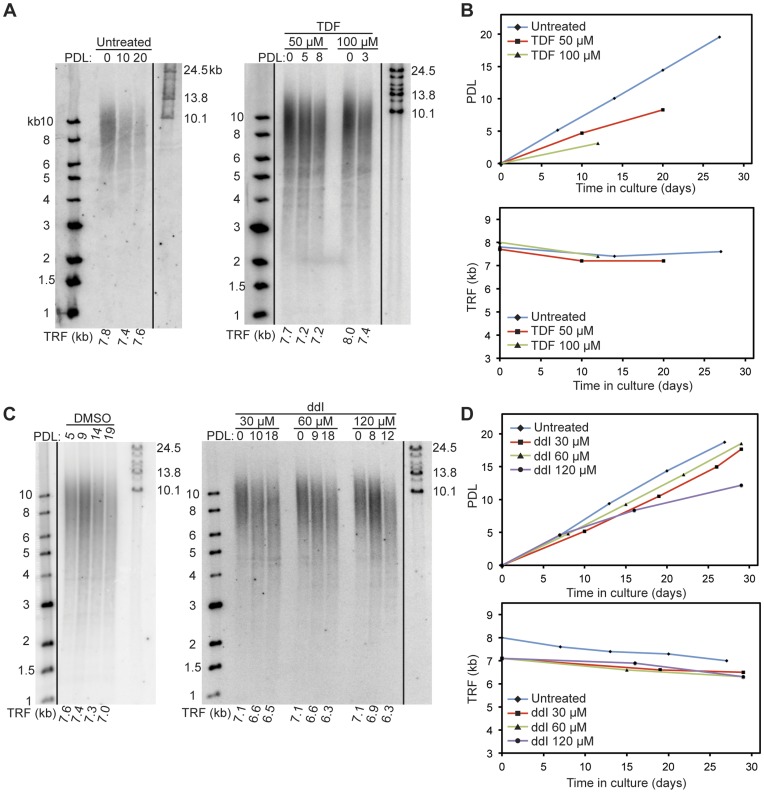
Continuous treatment of HT29 cells with the adenosine analogs TDF and ddI causes observable telomere shortening. A. TRF blots of untreated (left), TDF-treated (right) HT29 cells. PDL at which TRF was analyzed is shown above each lane. Molecular mass markers are shown at left and right of gel images. Each TRF smear was quantified as a weighted average and is shown below each lane. **B.** Growth curves and telomere maintenance dynamics of HT29 cells treated continuously with TDF. The growth curve and TRF dynamics of untreated HT29 cells (solid blue line) is plotted for comparison. There were moderate levels of telomere length loss in both the lower (50 µM) and higher (100 µM) TDF doses. However, these observations are marred by TDF cellular toxicities that prevent longer-term TRF analysis. **C.** TRF blots of DMSO-treated (control vehicle, left) and ddI-treated (right) HT29 cells. **D.** Growth curves and telomere maintenance dynamics of HT29 cells treated continuously with ddI. The growth curve and TRF dynamics of DMSO-treated HT29 cells (solid blue line) is plotted for comparison. Moderate telomere length loss over time was observed in all three doses (30 µM, 60 µM and 120 µM) of ddI treatments.

Analysis of primer extension assay data was performed with GraphPad Prism (v. 4.0b, GraphPad Software, Inc.). Student’s T-test for independent samples was used to compare means between each NNRTI and the DMSO control. The difference between means was considered statistically significant if the probability of making a type I error was less than 5% (i.e., p<0.05).

### Additional Methods and Procedures can be Found in the Supplementary Information Section

## Results

### In vitro Measurement of Telomere Repeat Synthesis by the Conventional Telomerase Primer Extension Assay

The telomerase primer extension assay reports single nucleotide incorporation and repeat synthesis by the telomerase enzyme *in vitro*. Prior to our studies, we performed a series of quality control experiments to ensure that experimental conditions accurately reflected the biochemical activities of telomerase.

**Figure 8:**
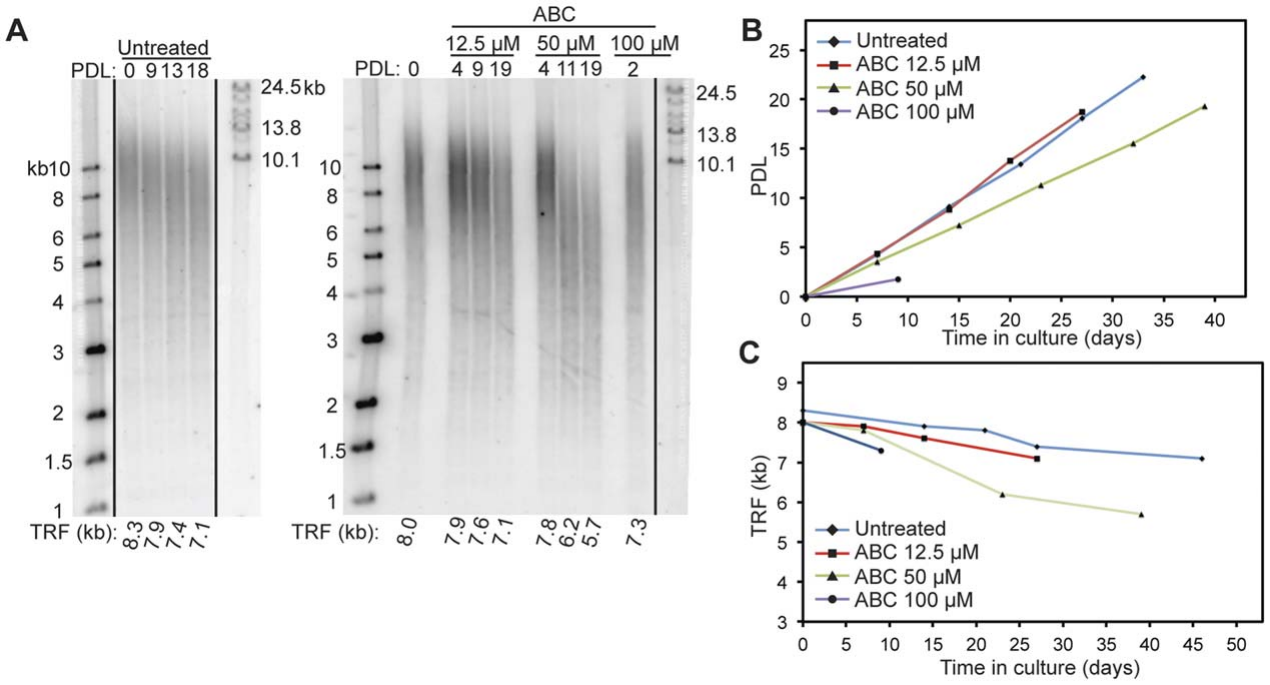
Continuous treatment of HT29 cells with the guanosine analog ABC causes telomere shortening. A. TRF blots of untreated (left) and ABC-treated (right) HT29 cells. PDL at which TRF was analyzed is shown above each lane. Molecular mass markers are shown at left and right of gel images. Each TRF smear was quantified as a weighted average and is shown below each lane. **B.** Growth curves of HT29 cells treated continuously with ABC. The growth curve of untreated HT29 cells (blue line) is plotted for comparison. **C.** Telomere length maintenance dynamics. The TRF dynamics of untreated HT29 cells (blue line) is plotted for comparison. Loss of telomere length is observed with the two lower doses (12.5 µM and 50 µM) of ABC treatments, with an apparent dose-response relationship. Cellular toxicity induced by the treatment with the highest (100 µM) ABC dose prevents longer-term TRF analysis, but substantial TRF loss was observed at PDL2.

To increase telomerase primer extension assay signals, we over-expressed recombinant telomerase RNA (TER) and TERT in 293HEK cells. Recombinant TERT was N-terminally tagged with three tandem repeats of the FLAG epitope. We compared the primer-extension activity profiles of whole-cell extracts from 293HEK over-expressing recombinant TER and TERT to recombinant telomerase from the same 293HEK cells partially immunopurified with anti-FLAG M2 antibodies. A comparison of the two activity profiles did not reveal any substantial changes in primer extension activity or repeat addition synthesis (Figure 1A). However, by removing other 293HEK extract components through immunopurification, the signal-to-noise ratios of the primer extension products were significantly improved. We therefore incorporated the immunopurification step in the standard activity assay procedure.

The immunopurification efficiency was determined by measuring the co-purified TER in the complex as a quantitative indicator of the holoenzyme copy number (both TERT and TER). Using quantitative RT-PCR [26], total TER copy numbers were compared before and after immunopurification (IP) of the transiently transfected whole-cell lysate from 293 HEK. Average IP efficiency was approximately 21% (Supplementary Figure 1A). Based on these measurements, the telomerase holoenzyme input into this primer extension assay was estimated to be 8×10^10^ copies per 40 µL reaction.

We also performed a primer extension assay with different primer permutations to ensure that the purified telomerase enzyme accurately copied the TER template and generated the expected pattern of telomeric DNA repeats. We synthesized a series of six 18 nt primers, each primer ending with a different nucleotide of the telomeric DNA repeat. Profiles of primer extension products using these six primer permutations were as expected. Each set of products reflected accurate template positioning, with precise repeat synthesis termination and translocation (Figure 1B). We concluded that recombinantly expressed and immunopurified telomerase faithfully reproduced native telomerase activity *in vitro*.

Assay reproducibility was tested in the presence of dideoxy nucleotide triphosphate (ddNTP) inhibitors, ddATP, ddTTP and ddGTP. However, ddCTP was not tested, as there is no cytidine residue in the 6 nt telomeric repeat. Dose-response curves of telomerase activity were estimated using four- or five-fold serial dilutions of inhibitors. Telomerase catalysis was inhibited in a dose-dependent manner with the addition of ddNTPs. For each ddNTP inhibitor, telomerase primer extension products were truncated at the corresponding complementary nucleotide on the template, corroborating the fidelity of telomerase catalysis in our *in vitro* system. Using the same immunopurified recombinant telomerase enzyme source on different days, inter-assay variability was assessed with at least three repeated measurements (Supplementary Figure 1B, Supplementary Table 1). We concluded that the telomerase primer extension assay accurately reflects telomerase catalysis *in vitro* and is sufficiently robust for the measurement of enzyme inhibition by nucleotide analogs.

**Table 3:** Comparison of inhibitory potencies of selected NRTIs on different families of nucleic acid polymerases.

Drugs	Polymerases	Selection§/Discrimination*Factors	References
AZT-TP	TERT	4.4*	This study
	HIV-RT	2.5§	[35]
	DNA polymerase gamma	10,000,000§	[35]
	DNA polymerase beta	2,600§	[35]
d4T-TP	TERT	2.4*	This study
	HIV-RT	2.2–4.5§	[45]
	DNA polymerase gamma	73§	[46]
TFV-DP	TERT	5.5*	This study
	HIV-RT	6§	[35]
	DNA polymerase gamma	10,800§	[35]
	DNA polymerase beta	40§	[35]
CBV-TP	TERT	18*	This study
	HIV-RT	10–30§	[47]
	DNA polymerase gamma	892,857§	[48]

§ Selection Factor = (Efficiency incorporation_dNTP_) ÷ (Efficiency incorporation_NRTI_).

### Telomerase Activity is Effectively Inhibited by Thymidine Analogs Zidovudine (AZT) and Stavudine (d4T)

To measure telomerase catalysis in the presence of thymidine analogs, we performed primer extension assays with an 18 nt telomere oligonucleotide primer ending with TTA (TTA-primer, Figure 2A). With this primer permutation, telomerase incorporates a single radiolabeled guanosine before reaching the end of the TER template region. At this point, telomerase can either dissociate from the G-extended primer, or translocate to the beginning of the TER template for the next round of repeat synthesis. Accordingly, in the absence of enzyme inhibitor, accumulation of primer extension products can be observed at regular 6 nt intervals, starting at TTA-primer+1, and continuing at +7, +13 and so on [27], [28]. As the signal from the first guanosine residue incorporation (TTA-primer+1) was not predicted to change in the presence of inhibitory thymidine and adenosine analogs, it was used as an internal control for the normalization of enzyme activity. Truncated primer extension products are predicted to accumulate at TTA-primer+4 and +5 for thymidine analogs (Figure 2A and 2B).

We measured the inhibitory potency of dideoxy thymidine triphosphate (ddTTP) in the telomerase primer extension assay as a positive control for thymidine analog inhibitors. Telomerase processivity decreased, and truncated primer extension products became evident in a dose-dependent manner at TTA-primer+4 and +5 positions. Telomerase catalysis was measured as a sum of both truncated and normal-length products, and normalized to the internal control (TTA-primer+1 signals). For our *in vitro* experiments, we used the fully activated (phosphorylated) forms of NRTIs. Addition of AZT-triphosphate (AZT-TP) and d4T-TP produced a product profile similar to ddTTP, confirming their function as chain-terminating thymidine analogs (Figure 2B). Our dose-response primer extension assays allowed us to estimate the inhibitory potencies (IC50s) of NRTIs against telomerase catalysis. These measurements, however, should be interpreted in context of concentrations of the input competitor nucleotides. We elected to express this relationship as the Discrimination Factor (DF), defined as the fold increase in ddNTPs or NRTIs concentration required for 50% telomerase inhibition (IC50) above that of each of the respective endogenous competitor dNTP concentrations (DF = IC50_NRTI/ddNTP_/[dNTP_competitor_]). A comparison of the DFs for ddTTP, AZT-TP and d4T-TP, under our assay conditions reveals very similar DF values for ddTTP and d4T (3.0 versus 2.4), and a slightly higher value for AZT-TP (4.4; Table 1 and Figure 2C). We concluded that telomerase catalysis is efficiently inhibited by AZT-TP and d4T-TP *in vitro*, consistent with their roles as chain-terminating thymidine analogs in the context of inhibition of HIV RT.

### Telomerase Activity is Inhibited by the Adenosine Analogs Tenofovir (TFV) and Didanosine (ddI)

We tested the active forms of tenofovir (TFV), an acyclic adenosine monophosphate analog, and didanosine (ddI). ddI is biotransformed to its active metabolite, dideoxyadenosine triphosphate (ddATP), which is the same molecule as our comparison standard [29]. Both adenosine analogs were investigated using the same primer extension assay as described for the thymidine analogs (Figures 2A and 3A). However, in the presence of adenosine analogs, truncated products are expected to accumulate at the TTA-primer+6 position, not the TTA-primer+4/+5 positions as was the case for thymidine analogs.

Incubation of primer extension reactions with the positive control ddATP (also the active form of ddI) resulted in the dose-dependent appearance of truncated TTA-primer+6 products. Truncated TTA-primer+6 products were also observed upon incubation with TFV-diphosphate (TFV-DP, the activated form of TFV), confirming its role as a chain-terminating adenosine analog (Figure 3B). However, in contrast to ddATP, we also observed a loss of the first guanosine incorporation at the highest TFV-DP concentration (Figure 3B, TTA-primer+1 band, 2300 µM lane). This suggests that TFV-DP has a mixed inhibition mode, and could also be incorporated by telomerase as a guanosine analog at high concentrations. However, its relative potency indicated that TFV-DP primarily acts as an adenosine analog.

A comparison of the DFs for ddATP and TFV-DP shows that these values differ by nearly one order of magnitude (DF_ddATP_ 0.63 compared to DF_TFV-DP_ 5.5) (Table 1 and Figure 3C). We concluded that telomerase catalysis is inhibited by TFV-DP *in vitro* with less potency than ddATP (ddI), and that TFV-DP may have a mixed mechanism of inhibition.

### Telomerase Activity is Inhibited by the Guanosine Analog Abacavir (ABC)

Abacavir (ABC) is the sole ddGTP analog in the NRTI family of antiretroviral agents. For ddGTP and its analogs, we used a different primer extension assay set-up to measure telomerase catalysis. An 18 nt telomere oligonucleotide primer ending with AGG (AGG-primer, Figure 4A) was used in the presence of radiolabeled dTTP, and dATP was withheld from the reaction. Under these assay conditions, telomerase incorporates a single guanosine, followed by the incorporation of two radiolabeled thymidines. Catalysis was expected to terminate after the addition of two thymidine residues due to the absence of dATP. Thus, under these conditions, normal catalysis terminates at the AGG-primer+3 position. In the presence of chain-terminating guanosine analogs or ddGTP, the incorporation of radiolabeled thymidine is expected to diminish, leading to the decrease in the AGG-primer+3 radioactive signal (Figure 4A). Dose-dependent loss of the radioactive signal was monitored as a functional readout of ddGTP incorporation and telomerase inhibition. This modification of our standard primer extension assay was necessary to avoid measuring the effects of chain-terminating guanosine analogs on primer-product translocation. The dissociation of primer-product at the end of the template synthesis (ending with a guanosine) could complicate the analysis of NRTI potency in nucleotide addition competition.

Incubation of primer extension reactions with the positive control ddGTP resulted in the dose-dependent disappearance of AGG-primer+3 products. Addition of carbovir-TP (CBV-TP, the active form of Abacavir, ABC) to the reaction resulted in a similar AGG-primer+3 product profile, in agreement with its role as a guanosine analog chain terminator (Figure 4B). Non-linear regression analysis of the dose-response relationships (Figure 4C) resulted in a DF of 1.6 for ddGTP and a DF of 18 for CBV-TP (Table 1). Our data indicate that the active form of ABC inhibits telomerase catalysis effectively *in vitro*, albeit with less potency than ddGTP.

### Telomerase Catalysis is not Affected by the NNRTIs Nevirapine and Efavirenz

Unlike NRTIs, which are competitive RT inhibitors, non-nucleoside reverse transcriptase inhibitors (NNRTIs), including nevirapine (NVP) and efavirenz (EFV), inhibit HIV RT non-competitively via enzyme binding outside the catalytic cleft [30]. We utilized our robust telomerase primer extension assay to determine whether these agents affect telomerase by binding to its RT domain, like their inhibition of HIV RT.

To measure telomerase catalysis in the presence of NVP and EFV, we used an 18 nt telomeric oligonucleotide primer ending in GGG (GGG-primer) and the full complement of three deoxynucleotide triphosphates (dATP, dTTP and dGTP), including radiolabeled dGTP. We tested both NNRTIs at 1 mM (in 5% DMSO) and 4 mM (in 10% DMSO) (Figure 5A–C). We also conducted dose-response experiments for NVP and EFV, and did not observe any changes in telomerase catalysis, even with concentrations as high as 10 mM (data not shown). We concluded that, under these conditions, telomerase catalysis is not inhibited *in vitro* by either of the NNRTIs tested.

### Telomere Length Maintenance in Cultured Human Cells Treated with NRTIs and NNRTIs

We measured the effects of long-term NRTI and NNRTI exposure on telomere length maintenance using the HT29 human colorectal adenocarcinoma cell model (Table 2). HT29 has robust telomerase activities, as measured by the PCR-based telomerase activity assay [31]. HT29 cells were treated with a minimum of two concentrations of NRTIs or NNRTIs. Cell proliferation and growth rate was monitored continuously. Long-term treatment of HT29 cells with AZT is known to cause telomere length attrition [9], [10], [11], [13]. Using the terminal restriction fragment (TRF) assay, we confirmed substantial inhibitory effects of AZT on telomere length maintenance in HT29 cells (Figure 6A and 6B). Mean telomere length was determined as a weighted average with reference to DNA standards. At 62.5 and 125 µM AZT, we observed 1.9 kb and 2.3 kb losses in mean telomere length, respectively, over 12 population doubling levels (PDL) in continuous culture. At 125 µM AZT, the rate of telomeric DNA loss was 192 bp/PDL, suggesting that telomerase activity was completely inhibited at this dose.

Continuous treatment of HT29 cells with d4T also resulted in a dose-dependent loss of telomeric DNA (Figures 6C and 6D). Treatment with 80 µM or 160 µM of d4T resulted in 1.5 kb and 3.7 kb losses in mean TRF signals over 21 and 18 PDL, respectively. Unexpectedly, treatment with tenofovir disoproxil fumarate (TDF, the prodrug of tenofovir) resulted in excessive cell death, and we were unable collect any cell populations beyond 8 PDL for the 50 µM treatment groups and 3 PDL for the 100 µM treatment group (Figures 7A and 7B). This is in contrast with the low cytotoxicity reported with this agent in other cultured cell models [32]. At these early PDLs, the cumulative TRF loss was expected to exceed the inherent variation of our assay. Thus, we cannot deduce the effects of TDF on telomere maintenance from this experiment. Continuous treatment with ddI at 30 and 60 µM showed no observable loss of TRF signals up to 18 PDL (Figures 7C and 7D). At 120 uM, ddI showed a slight decrease (0.8 kb) in TRF size at 12 PDL. However, treatment cytotoxicity prevented us from collecting cells beyond this time point, and prevented us from confirming the effects of ddI on telomere maintenance.

Treatment with a high dose of ABC (100µM) had a profound cytotoxic effect on HT29 cells, and we were not able to collect cells beyond 2 PDL following drug treatment (Figure 8). However, with lower doses of ABC (12.5 µM and 50 µM), we did observe a dose-dependent loss of TRF signal (0.8 kb and 2.1 kb, respectively) over 19 PDL. In our experiments, we also included continuous treatment with Lamivudine (3TC), a cytidine analog not expected to affect telomere synthesis by telomerase, since the telomeric repeat lacks cytidine. The inclusion of 3TC as a negative control tested whether inhibition of other DNA polymerases could affect telomere maintenance independently of telomerase. As expected, we did not observe any loss of TRF signals over 19 PDL of continuous treatment with 80 µM of 3TC (Supplementary Figure 2), implying that telomere length changes measured in NRTI-treated cells are specific to telomerase inhibition effects under these conditions. Finally, continuous treatment with the two NNRTIs, EFV and NVP, did not result in significant losses of TRF signal, despite treatment toxicity associated with high doses of EFV (Supplementary Figure 3). This corroborated our *in vitro* observation that NNRTIs did not affect telomerase catalysis.

## Discussion

Using the *in vitro* primer extension assay for telomerase activity, we demonstrated that all NRTIs in current clinical usage inhibit human telomerase, albeit with different potencies. AZT-TP and d4T-TP were the most potent inhibitors of telomerase relative to their ddNTP counterpart, ddTTP. CBV-TP was the least potent telomerase inhibitor relative to ddGTP, while the TFV-DP/ddATP comparison showed intermediate inhibitory potency. There was general agreement between our *in vitro* and cell culture studies of NRTI effects on telomere synthesis, although in some instances, the profound toxicity of high concentrations of several NRTIs likely affected the correlation between the two experiments. In any cell culture system, effects of telomerase inhibition manifest as a gradual loss of telomere length, which is only evident in the TRF assay after sufficient time has passed (10–20 PDL when telomerase is inhibited completely). With the highest doses of ABC and TDF used in our studies, no cells survived beyond PDL 2 and 3, respectively. This high cellular toxicity is unexpected for TDF, as early toxicity studies have boasted a good safety profile for this drug [32]. In these cases, even if telomerase were completely inhibited, the lack of cells surviving for a sufficient time would prevent us from observing the full effect of telomere length attrition caused by enzyme inhibition. Additionally, under extreme selective pressure, transformed cells with unstable genomes can induce genetic changes much more readily. Pharmacokinetic changes caused by genetic modifications that increase tolerance to NRTI toxicity could effectively reduce intracellular concentrations of the active form of these drugs [33], [34]. Although telomerase itself might maintain its sensitivity to the NRTI, changes in the effective intracellular concentration of the active drug could cause a particular NRTI to appear less potent, as indicated by its effect on telomere length maintenance.

When comparing our findings to reports on the biochemical properties of these agents against the HIV-RT, we found that neither TERT, nor HIV RT, have a high level of discrimination against TFV-DP [35] or CBV-TP [36] *in vitro* (Table 3). In contrast, DNA polymerases preferentially select for the natural substrates dATP and dGTP, compared to TFV-DP and CBV-TP, respectively. Kinetic experiments indicate that HIV RT incorporates d4T-TP as efficiently as dTTP and that AZT-TP less efficiently than both d4T-TP and dTTP [37]. Although we did not perform detailed kinetic analyses of telomerase in the presence of AZT and d4T, our primer extension assay data suggest a similar trend. In summary, our data support the notion that all tested NRTIs could be incorporated into telomeric DNA by telomerase, resulting in chain termination. Although the telomeric sequence is non-coding, sequence-specific binding of the shelterin complex could be disrupted, even with a single mismatched telomeric sequence [38]. Uncapped or poorly capped telomeres–caused by changes in telomeric repeat sequences–are recognized as signals of DNA damage, leading to the temporary halt of cell proliferation, or cell death [38], [39]. Thus, NRTI incorporation into telomeres could contribute to premature cell death beyond its role in accelerated telomere attrition [40]. This is an important consideration when determining the off-target effects of these agents in human cells. Accelerated telomere attrition is associated with diminished cellular renewal capacity. Loss of this regeneration capacity could contribute, in part, to the underlying cause for the observed premature age-related co-morbidities in HIV-infected patients [41], [42]. Furthermore, our results would suggest that NNRTI and selected NRTIs, perhaps such as C analogs, would be less likely to exert long-term effects on telomeres and possibly tissue regeneration.

This study provides the impetus to conduct additional investigations on the effects of NRTIs on telomerase activity and telomere maintenance *in vitro* and *in vivo*. Future in vitro studies on the impact of NRTIs on telomere maintenance in human cells should focus on primary human cells. Relevant cell types, such as human stem cells, would provide an ideal model for these experiments, but their maintenance in culture for extended periods of time may pose a significant challenge. In future studies, chemotherapeutic treatment combinations could be designed based on their current clinical usage to observe the extent of their synergistic effect on telomere length maintenance. To generate a more comprehensive pattern of NRTI activity across a spectrum of tissue types, a panel of human cells comprising different tissues and cell types could be used in a screen for telomerase inhibition.

The advent of Highly Active Antiretroviral Therapy (HAART) 15 years ago greatly reduced mortality and morbidity in HIV-infected individuals. Together with protease inhibitors, NRTI and NNRTI are first-line ARVs, the cornerstones of HAART. Once started, HAART is usually life-long. New data indicate that increased HAART use among HIV-infected individuals has reduced HIV transmission rates [43], prompting a call for earlier induction and increased use of HAART in all HIV cases [44]. Given our results, it is prudent to conduct longitudinal or prospective investigations of the effects of long-term HAART with NRTIs.

## Supporting Information

Figure S1 **IP efficiency and reproducibility of the primer extension assay in the presence of ddNTPs.** **A.** Determination of the efficiency of IP telomerase from 293HEK cells transiently transfected with 3×FLAG TERT and TER using quantitative RT-PCR. Arrows under gel image roughly illustrate the point at which the endogenous TER is in equal quantity compared to its competitor RNA. An example calculation for one transfection is shown. **B.** Reproducibility of the primer extension assay in the presence of ddNTPs.
(TIF)

Figure S2 **Continuous treatment of HT29 cells with the cytidine analog 3TC does not affect telomere maintenance.** **A.** Growth curve of HT29 cells treated continuously with 3TC. The growth curve of untreated HT29 cells (blue line) is plotted for comparison. **B.** Telomere maintenance dynamics in cells shown in **A.** **C.** TRF blots of untreated (left) and 3TC-treated (right) HT29 cells. PDL at which TRF was analyzed is shown above each lane. Molecular mass markers are shown at left and right of gel images. Each TRF smear was quantified as a weighted average and is shown below each lane.
(TIF)

Figure S3 **Continuous treatment of HT29 cells with the NNRTIs NVP and EFV does not affect telomere maintenance. A.** Growth curves of HT29 cells treated continuously with NVP (left) or EFV (right). The growth curve of untreated HT29 cells (blue line) is plotted for comparison. **B.** Telomere maintenance dynamics in cells shown in **A.** **C.** TRF blots of untreated HT29 cells. PDL at which TRF was analyzed is shown above each lane. Molecular mass markers are shown at left and right of gel images. Each TRF smear was quantified as a weighted average and is shown below each lane. **D–E.** TRF blots of NVP-treated (**D**) or EFV-treated (**E**) HT29 cells.
(TIF)

Table S1 **Assay setup and reproducibility for testing chain-terminating thymidine, adenosine, and guanosine analogs against telomerase.**
(DOCX)

Materials and Methods S1 (DOCX)
