# Supplementary figures and images for: In Vitro and Ex Vivo Inhibition of Human Telomerase by Anti-HIV Nucleoside Reverse Transcriptase Inhibitors (NRTIs) but Not by Non-NRTIs
Source: PLoS One. 2012 Nov 15;7(11):e47505. doi: 10.1371/journal.pone.0047505 (PMC3499584; doi:10.1371/journal.pone.0047505)

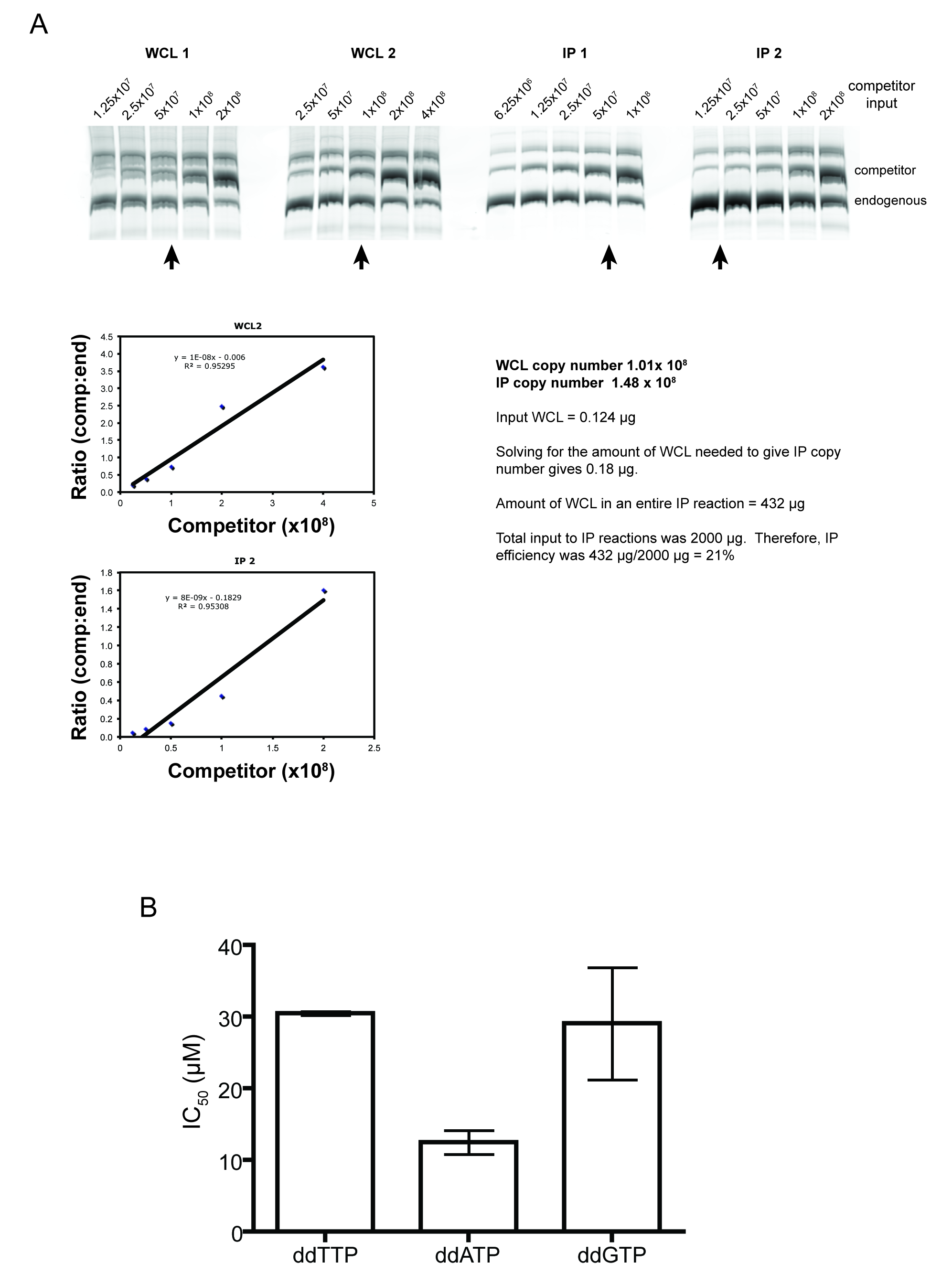

Supplement: Figure S1 — IP efficiency and reproducibility of the primer extension assay in the presence of ddNTPs. A. Determination of the efficiency of IP telomerase from 293HEK cells transiently transfected with 3×FLAG TERT and TER using quantitative RT-PCR. Arrows under gel image roughly illustrate the point at which the endogenous TER is in equal quantity compared to its competitor RNA. An example calculation for one transfection is shown. B. Reproducibility of the primer extension assay in the presence of ddNTPs. (TIF) [file pone.0047505.s001.tif]

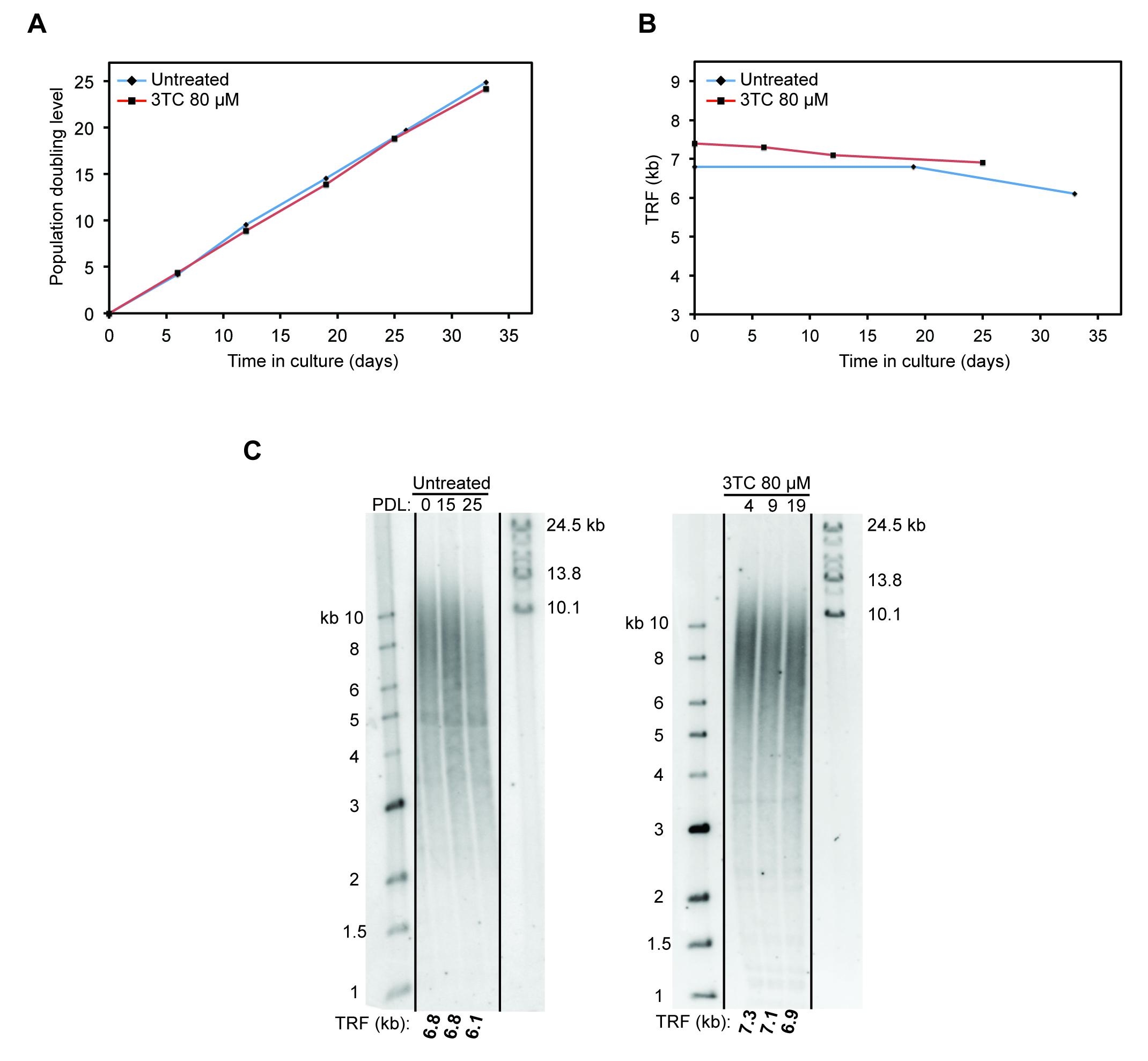

Supplement: Figure S2 — Continuous treatment of HT29 cells with the cytidine analog 3TC does not affect telomere maintenance. A. Growth curve of HT29 cells treated continuously with 3TC. The growth curve of untreated HT29 cells (blue line) is plotted for comparison. B. Telomere maintenance dynamics in cells shown in A. C. TRF blots of untreated (left) and 3TC-treated (right) HT29 cells. PDL at which TRF was analyzed is shown above each lane. Molecular mass markers are shown at left and right of gel images. Each TRF smear was quantified as a weighted average and is shown below each lane. (TIF) [file pone.0047505.s002.tif]

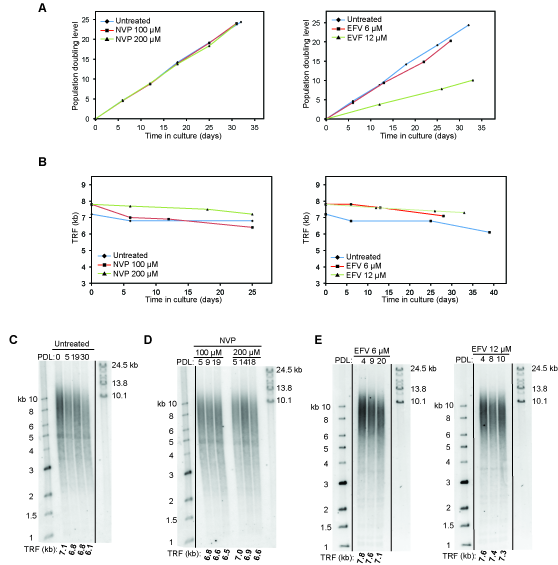

Supplement: Figure S3 — Continuous treatment of HT29 cells with the NNRTIs NVP and EFV does not affect telomere maintenance. A. Growth curves of HT29 cells treated continuously with NVP (left) or EFV (right). The growth curve of untreated HT29 cells (blue line) is plotted for comparison. B. Telomere maintenance dynamics in cells shown in A. C. TRF blots of untreated HT29 cells. PDL at which TRF was analyzed is shown above each lane. Molecular mass markers are shown at left and right of gel images. Each TRF smear was quantified as a weighted average and is shown below each lane. D–E. TRF blots of NVP-treated (D) or EFV-treated (E) HT29 cells. (TIF) [file pone.0047505.s003.tif]
