# Supplementary material for: In Vitro and Ex Vivo Inhibition of Human Telomerase by Anti-HIV Nucleoside Reverse Transcriptase Inhibitors (NRTIs) but Not by Non-NRTIs
Source: PLoS One. 2012 Nov 15;7(11):e47505. doi: 10.1371/journal.pone.0047505 (PMC3499584; doi:10.1371/journal.pone.0047505)
Supplement: Table S1 — Assay setup and reproducibility for testing chain-terminating thymidine, adenosine, and guanosine analogs against telomerase. (DOCX) [file pone.0047505.s004.docx]

**Table S1.** Assay setup and reproducibility for testing chain-terminating thymidine, adenosine, and guanosine analogs against telomerase.

| **ddNTP** | **dTTP (μM)** | **dATP (μM)** | **dGTP (μM)** | **Labeled dNTP*** | **IC_50_ ± SD (μM)** | **Coefficient of Variation (%)** |
| --- | --- | --- | --- | --- | --- | --- |
| **ddTTP** | 10 | 1000 | 10 | [α-^32^P]dGTP | 30.5 ± 0.4 | 1.5 |
| **ddATP** | 1000 | 20 | 10 | [α-^32^P]dGTP | 12.5 ± 2.9 | 23.2 |
| **ddGTP** | 10 | - | 20 | [α-^32^P]dTTP | 29.1 ± 7.8 | 27.0 |

*labeled dNTPs used in 200 nM final concentration.
