# Supplementary material for: In Vitro and Ex Vivo Inhibition of Human Telomerase by Anti-HIV Nucleoside Reverse Transcriptase Inhibitors (NRTIs) but Not by Non-NRTIs
Source: PLoS One. 2012 Nov 15;7(11):e47505. doi: 10.1371/journal.pone.0047505 (PMC3499584; doi:10.1371/journal.pone.0047505)
Supplement: Materials and Methods S1 — (DOCX) [file pone.0047505.s005.docx]

**Materials and Methods S1**

*Cell culture*

The human colon cancer cell line HT29 (ATCC, Manassas, VA) was cultured in T25 flasks in Dulbecco’s Modified Eagle’s Medium (DMEM) supplemented with 5% fetal bovine serum (FBS) and 100U each penicillin and streptomycin. Cells were maintained at 37°C in 5% CO2. Cells were harvested with 0.25% trypsin-EDTA at 85% confluency.

NRTIs and NNRTIs were dissolved in either PBS or dimethyl sulfoxide (DMSO) and stored at 20°C. Diluted working aliquots were prepared for cell culture experiments, and drugs were added, as 0.1% (d4T, ABC, ddI, NVP, EFV, 3TC), 0.17% (AZT), or 0.5% (TDF) of the final culture media volume. As controls, PBS and DMSO were added to media as 0.5% and 0.1% of the final volume, respectively. d4T, AZT, 3TC, and TDF were dissolved in PBS, while NVP, EFV, ABC, and ddI were dissolved in DMSO.

*Transient Transfection of 293 HEK cells*

293 human embryonic kidney cells (ATCC **CRL-1573,** Manassas, VA) were maintained in T175 flasks in DMEM supplemented with 5% FBS and 100U each of penicillin and streptomycin at 37°C in 5% CO_2_. Transient transfection of 293 HEK cells was performed using the calcium phosphate mediated transfection method [1]. Upon reaching 60% confluency, 293HEK cells were harvested and seeded on 150 cm plates, with a density of 1x10^7^ cells/plate. Transient transfection was performed 24 h after seeding with the two mammalian expression plasmids: 3X FLAG hTERT in pcDNA3.1 and U3-hTER in pBS (both kindly provided by Kathleen Collins’ laboratory). Media was changed 24 h after transfection and cells were harvested 48 h later. Harvested cells were washed once with 1X PBS (1 mL), then stored at -80°C for subsequent whole cell lysate preparation.

*Extraction of whole cell lysate and immunopreciptation*

Harvested cells were washed once in 1 X PBS (1 mL) and centrifuged at 5000 rpm for 1 min at 4°C in 1.5 mL microcentrifuge tubes. After washing, cells were extracted for whole cell lysate with three repeats of freeze-thaw lysis, as described [2]. Protein concentration of WCL was determined using the Bradford method (Bio-Rad Laboratories Inc., Hercules, CA), according to the manufacturer’s protocol. Immunopurification of FLAG-tagged hTERT was essentially as described [3]. After the third wash, reactions were centrifuged at 5000 rpm for 30 s at 4°C, and beads were resuspended in 130 μL IP wash buffer (20 mM HEPES pH 8, 2 mM MgCl_2_, 0.2 mM EGTA, 1 mM DTT, 0.1 mM PMSF,10% glycerol, 0.1% NP-40), then stored at -80°C until use.

*Genomic DNA isolation and Terminal Restriction Fragment (TRF) assay*

Genomic DNA was obtained using the QIAamp DNA Blood Mini Kit (Qiagen) and resuspended in 200 μL Elution Buffer (Qiagen). Genomic DNA (approximately 20-30 μg) was digested overnight at 37°C with 2 units each of HinfI and RsaI (New England Biolabs,Ipswich, MA). Digested DNA was precipitated and quantified with the QuantIT PicoGreen assay (Sigma-Aldrich, Oakville, ON) according to the manufacturer’s protocol. Samples were analyzed using either the Typhoon Trio Imager (GE Healthcare Lifesciences) fitted with the appropriate laser (488 nm), or the SynergyMX plate reader (BioTek) with the correct light source. Two micrograms of restriction enzyme-digested genomic DNA was resolved on 0.5% agarose gel along with ^32^P radiolabeled DNA markers (New England Biolabs and Fermentas, Burlington, ON). In gel hybridization was done essentially as described [4]. TRF signals were imaged with storage phosphor screens (GE Healthcare Lifescience) using the Typhoon Trio Imager.

Signal quantification was performed using the ImageQuant (v. 5.2) software. TRF length was determined as a weighted average. The entire gel image was divided into horizontal segments corresponding to the size markers. To obtain the TRF score for each sample, the optical density (OD) of each horizontal segment was multiplied by the length corresponding to the respective segment, summed, then divided by the sum of the OD of all segments (i.e., TRF=Σ(ODi*Li)/ΣOdi, where ODi is optical density at interval i, and Li is average length at interval i). TRF lengths are reported as kb to one decimal place.

*Competitive RT-PCR for TER Quantification*

Total RNA was prepared from WCL and IP samples using Trizol (Invitrogen). In separate reactions, 10 μL of each RNA sample together with the calculated number of copies of synthetic input competitor RNA molecules were reverse transcribed with a TER reverse primer (5'-GCC TGG GAG GGG TGG TG-3'). Ten microliters of the RT reaction were taken for PCR with the addition of TER forward primer (5'-Cy5-GCC TGG GAG GGG TGG TG-3'). The PCR conditions were as follows: 30 cycles of 95°C for 30 sec (denaturing), 55°C for 30 sec and 72°C followed by a single extension step of 72°C for 5 min.

PCR amplicons were resolved through non-denaturing polyacrylamide gel electrophoresis (10%) and visualized by laser scanning at 650 nm using the Typhoon Trio (GE Healthcare Sciences). Bands corresponding to the endogenous TER amplicons (122 bp) and the competitor amplicon (142 bp, with a 20-nt non-specific DNA insertion internally into the endogenous TER amplicon). Competitive PCR product signals were quantified using ImageQuant (v. 5.2, GE Healthcare Life Sciences). A ratio of endogenous amplicon:competitor amplicon was calculated based on the intensity of these bands within each lane. The endogenous amplicon:competitor amplicon ratio was plotted against input competitor RNA copy number and linear regression was performed. The linear equation was used to determine the copy number of competitor RNA required to generate an endogenous amplicon:competitor amplicon ratio of 1. The TER copy numbers found in the WCL and IP samples for each independent transient transfection experiment were then used to determine the IP efficiency.

**Supporting Information References**

1. Sambrook J, Russell DW (2001) Molecular cloning : a laboratory manual. Cold Spring Harbor, N.Y.: Cold Spring Harbor Laboratory Press.

2. Mitchell JR, Wood E, Collins K (1999) A telomerase component is defective in the human disease dyskeratosis congenita. Nature 402: 551-555.

3. Robart AR, Collins K (2010) Investigation of human telomerase holoenzyme assembly, activity, and processivity using disease-linked subunit variants. J Biol Chem 285: 4375-4386.

4. Wong JM, Collins K (2006) Telomerase RNA level limits telomere maintenance in X-linked dyskeratosis congenita. Genes Dev 20: 2848-2858.
